# COVID-19 underreporting and its impact on vaccination strategies

**DOI:** 10.1186/s12879-021-06780-7

**Published:** 2021-10-28

**Authors:** Vinicius Albani, Jennifer Loria, Eduardo Massad, Jorge Zubelli

**Affiliations:** 1 Department of Mathematics, Federal University of Santa Catarina, Florianopolis, Brazil; 2 Instituto de Matemática Pura e Aplicada, Rio de Janeiro, Brazil; 3 Universidad de Costa Rica, San Jose, Costa Rica; 4 School of Applied Mathematics, Fundação Getúlio Vargas, Rio de Janeiro, Brazil; 5 School of Medicine, University of São Paulo and LIM01-HCFMUSP, São Paulo, Brazil; 6 Mathematics Department, Khalifa University, Abu Dhabi, UAE

**Keywords:** Underreported infections, Underreporting estimation, Vaccination strategies, Epidemiological models, Stable rates of hospitalization and death, Numerical simulation

## Abstract

**Background:**

Underreporting cases of infectious diseases poses a major challenge in the analysis of their epidemiological characteristics and dynamical aspects. Without accurate numerical estimates it is difficult to precisely quantify the proportions of severe and critical cases, as well as the mortality rate. Such estimates can be provided for instance by testing the presence of the virus. However, during an ongoing epidemic, such tests’ implementation is a daunting task. This work addresses this issue by presenting a methodology to estimate underreported infections based on approximations of the stable rates of hospitalization and death.

**Methods:**

We present a novel methodology for the stable rate estimation of hospitalization and death related to the Corona Virus Disease 2019 (COVID-19) using publicly available reports from various distinct communities. These rates are then used to estimate underreported infections on the corresponding areas by making use of reported daily hospitalizations and deaths. The impact of underreporting infections on vaccination strategies is estimated under different disease-transmission scenarios using a Susceptible-Exposed-Infective-Removed-like (SEIR) epidemiological model.

**Results:**

For the considered locations, during the period of study, the estimations suggest that the number of infected individuals could reach 30% of the population of these places, representing, in some cases, more than six times the observed numbers. These results are in close agreement with estimates from independent seroprevalence studies, thus providing a strong validation of the proposed methodology. Moreover, the presence of large numbers of underreported infections can reduce the perceived impact of vaccination strategies in reducing rates of mortality and hospitalization.

**Conclusions:**

pBy using the proposed methodology and employing a judiciously chosen data analysis implementation, we estimate COVID-19 underreporting from publicly available data. This leads to a powerful way of quantifying underreporting impact on the efficacy of vaccination strategies. As a byproduct, we evaluate the impact of underreporting in the designing of vaccination strategies.

**Supplementary Information:**

The online version contains supplementary material available at 10.1186/s12879-021-06780-7.

## Background

Surveillance and notification systems in Public Health are subject to uncertainties that cause difficulties to estimate the morbidity and mortality rates affecting populations. Among the diverse causes of uncertainty two distinct levels of surveillance in Public Health should deserve special attention, under-ascertainment, when not all cases seek healthcare; and underreporting, a failure to adequately report symptomatic cases that have sought medical advice [1]. In the context of mortality, it is possible to identify the concepts of under-ascertainment and underreporting since both events are expected to happen in real systems of Public Health. Thus, in what follows, we unify under-ascertainment and underreporting as “underreporting”.

Underreporting cases of infectious diseases poses a major challenge in the analysis of their epidemiological characteristics and dynamical aspects. Without accurate numerical estimates it is difficult to precisely quantify the proportions of severe and critical cases, as well as the mortality rate [1]. Such estimates can be provided, e.g., by testing the presence of the virus. However, during an ongoing epidemic, such testing implementation is a daunting task.

Different strategies were proposed to estimate the true amount of COVID-19 cases. Some of these strategies are based on seroprevalence studies [2–6] that found seroprevalence proportions much larger than the reported accumulated cases in different periods of 2020 in Chicago and NYC, as well as across Denmark, Mexico, and the United States, respectively. Other works estimate underreported infections and deaths from the official reports in combination with different techniques. For example, in [7, 8] the authors consider the excess of deaths caused by respiratory infections in 2020 and found significant underreporting proportions in Brazil. The number of excess deaths is also estimated for England and Wales in [9]. Based on data provided by the World Heath Organization (WHO), the article [10] compares case-fatality risk measures for different countries to estimate underreporting. By using the data from South Korea as a benchmark, the authors in [11] built an underreporting estimation technique based on the predictions of a susceptible-infected-removed-type (SIR-type) model, that are adjusted using demographic data from different places. The work [12] proposes a Bayesian framework based on an SIR-type model to estimate the true case fatality ratio (CFR) and the corresponding underreporting using official reports from the Brazilian health authority. Similarly, in [13], the authors use a Susceptible-Exposed-Infected-Removed-type (SEIR-type) model to estimate the CFR and the underreported cases in Iran, based on data from WHO and the Iranian Health authority. Another application of an SIR-type model to estimate underreporting was performed in [14], using data from California and Florida. In [15], the authors estimate underreported deaths in Italy by comparing mortality data and making use of regression techniques, as well as demographic information. The work [16] proposes a machine learning algorithm to predict underreported infections for all the 50 states in the US and other countries, using official reports and the infection-fatality-rate estimated in [17] as the training dataset.

COVID-19 control is dependent, in complex non-linear ways, on several variables that include the incidence of infection, on non-pharmaceutical interventions like the use of masks and social distancing, the speed with which the vaccination can be implemented, and the efficacy of the available vaccines. The uncertainties and interactions between these variables make the use of mathematical models to quantify and optimise the effects of vaccination on the COVID-19 pandemic urgently needed [18]. Mathematical models, therefore, have played a key role in helping the understanding of COVID-19 dynamics as well as in determining the best decisions of mitigation strategies [19]. In this sense, models remain essential tools for evidence synthesis, planning and forecasting, decision analysis for COVID-9 control, as well as policymaking [20].

This work presents a methodology to estimate underreported infections based on approximations of the stable rates of hospitalization and death found using daily reports of infections, hospitalizations, and deaths, as well as testing data. As an important byproduct, we evaluate the impact of underreporting in the designing of vaccination strategies because the larger the number of unaccounted infections, the larger the chances of vaccinating an already immune individual. This can restrict the capability of vaccination in reducing hospitalizations and deaths, as simulated scenarios using an SEIR-like model [21] show. It is worth mentioning that, understanding such limitations is particularly important to help scientists and authorities addressing the politicization of the vaccination, the polemic around safety and efficacy of the vaccines, and the anti-vaccination campaigns that contribute to vaccination hesitancy and vaccination delay [22, 23].

## Methods

**Fig. 1:**
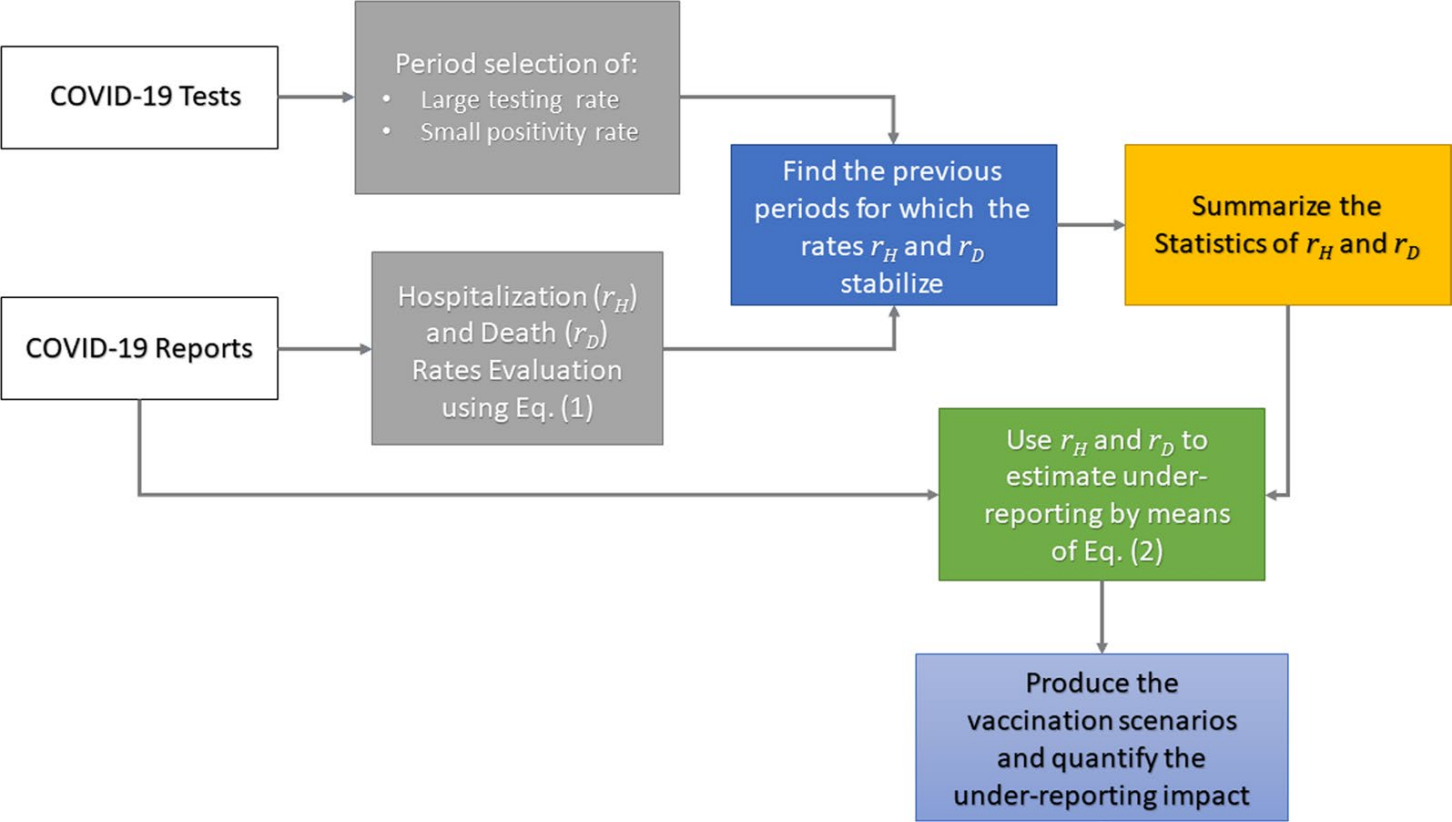
Methodological workflow for the underreporting quantification

This section starts by presenting how the stable rates of hospitalization and death are obtained. Then, the technique to estimate the potential underreporting of COVID-19 infections is introduced. Finally, a Susceptible-Exposed-Infected-Removed-like (SEIR-like) epidemiological model is proposed to quantify how underreporting may affect vaccination strategies. The schematic description for this methodology is shown in Fig. 1.

In order to find stable rates of hospitalization and death, we seek specific time periods when the daily rate of testing is sufficiently large with respect to the population size, and the number of positive tests is small enough. During such periods we evaluate daily empirical rates of hospitalization and death, looking for those whose rates fluctuate around some mean value. This is performed by means of an accurate data analysis producing different statistical indicators leading to the necessary correction. A schematic representation that summarizes the proposed methodology can be found in Fig. 1. We use time series of seven-day moving averaged reports from Chicago and New York City (NYC), in the US, the province of Buenos Aires (BA), in Argentina, and Mexico City (MC), in Mexico. Since COVID-19 severity strongly depends on age and gender [17, 24–27], we evaluate the above-mentioned rates accounting for demography to improve the estimation accuracy of the number of infections. The latter will be called corrections. These corrections are evaluated using the empirical rates of hospitalization and death as follows: For an observed rate of hospitalization or death, and a given day in the time series, we evaluate the corresponding infection number. For example, if for this day the reported hospitalization rate is one and the projected rate is one half, then, the correction is twice the reported infections.

### Rates of hospitalization and death

The procedure used to obtain stable rates of hospitalization and death is presented. For this, we firstly use daily total numbers of new infections, hospitalizations and deaths associated to COVID-19 in Chicago, then the reports for eight age ranges. The gender rates are also obtained, but the procedure is omitted, since it is similar to the case of age range.

*Stable Rates of Hospitalization and Death* Let us consider the time series of daily numbers of COVID-19 infections, hospitalizations, and deaths, as well as the performed and positive tests in Chicago, during the period 01-Mar-2020 to 24-Dec-2020, available at https://www.chicago.gov/city/en/sites/covid-19/home.html, and accessed on 28-Dec-2020. Accordingly to the aforementioned website, COVID-19 testing is focused on those individuals who have COVID-19 symptoms or who had contact with suspected or confirmed cases.

Since we are looking for the stable distribution of the hospitalization and death rates related to COVID-19 infections, to estimate underreporting we must find a period when the disease spread is stable and accurately observed. In other words, we consider the period when the number of tests performed daily is large, with respect to the population size, and the number of observed cases is small, with respect to the number of tests.

**Fig. 2:**
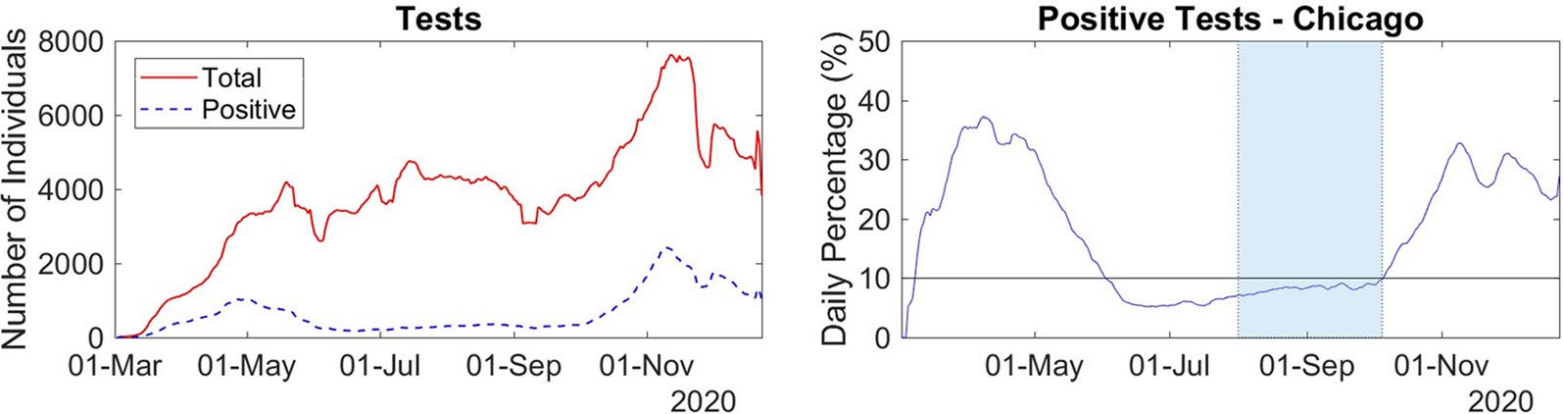
Left: Daily numbers of performed and positive tests of COVID-19 in Chicago. Right: The corresponding daily positive rate. The period is 01-Mar-2020 to 23-Dec-2020. The solid horizontal line represents a reference of 10%

Figure 2 presents the daily number of performed and positive tests, as well as the rate of positive tests for 01-Mar-2020 to 23-Dec-2020. From 02-Jun-2020 to 05-Oct-2020, the percentage of positive tests stayed below 10%, which may indicate that the number of tests performed during this period is much larger than the number of COVID-19 infections. In addition, during that period, the daily number of tests was above 3000, representing more than 0.1% of the population of Chicago estimated for 2020. Thus, we assume that during that period, the dataset from Chicago meets the necessary conditions to find the stable rates of hospitalization and death mentioned above.

Let us define the daily rates of hospitalizations and deaths. Firstly, let $\mathcal {I}$, $\mathcal {H}$ and $\mathcal {D}$ denote, respectively, the time series of daily reports of COVID-19 infections, hospitalizations, and deaths. Then, the rates of hospitalizations and death amongst infective individuals, as well as the death rate of hospitalized individuals are defined, respectively, as

1 $$\begin{aligned} r_H(t) = \dfrac{\mathcal {H}(t)}{\mathcal {I}(t-\tau _H)}, ~r_D(t) = \dfrac{\mathcal {D}(t)}{\mathcal {I}(t-\tau _D)}, ~ \text{ and } ~r_{DH}(t) = \dfrac{\mathcal {D}(t)}{\mathcal {H}(t-\tau _{DH})}. \end{aligned}$$

In [28], the mean time from onset to hospitalization is 1.2 day, so, we set $\tau _H =1$. The mean time from hospital admission to death is given by the sum of the mean times from hospitalization to admission to an intensive care unit (ICU), and from ICU admission to death were taken from References [29] and [30], respectively. Thus, we set $\tau _D = 12$ and $\tau _{DH} = 11$.

**Fig. 3:**
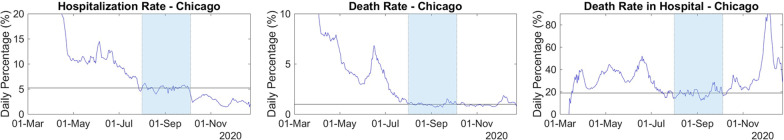
Daily rates of hospitalization (left), death (center) and death amongst hospitalized individuals (right) from 01-Mar-2020 to 23-Dec-2020, in Chicago. The dark area shows the period when the rate seems to stabilize, i.e., 01-Aug2020 to 05-Oct-2020. The horizontal solid lines represent the median of the rates observed during 01-Aug-2020 to 05-Oct-2020

Figure 3 presents the daily rates obtained using the formulas in Eq. 1 for the data from Chicago. During the period 01-Aug-2020 to 05-Oct-2020, all the three rates apparently stabilized around their mean values. In this period the series of tests performed daily is large enough and the number of positive tests is small enough, accordingly to our assumptions. Moreover, the outbreaks of March to May and October to December seem to not affect the reports on these dates. Therefore, we assume that the daily rate values obtained during such period are indeed observations of the stable rates of hospitalization, death, and death amongst hospitalized individuals. The median value and 90% confidence interval (90% CI) can be found in Table 1, in the *Citywide* row.

**Table 1:** Median values and 90% CI (numbers inside the parentheses) of the daily rates of hospitalization, death and death in hospital observed during the period 01-Aug-2020 to 05-Oct-2020 in Chicago

	Hospitalization rate	Death rate	Death rate in hospital
Age range			
0–17	1.12 (0.34–2.59)	0 (0–0)	0 (0–0)
18–29	2.05 (1.46–2.52)	0 (0–0.16)	0 (0–7.14)
30–39	2.69 (1.54–4.28)	0.22 (0–0.45)	7.69 (0–27.27)
40–49	4.22 (2.15–6.19)	0.31 (0–1.23)	8.89 (0–30.77)
50–59	6.45 (3.83–10.36)	1.11 (0.37–1.7)	15.69 (5.56–26.67)
60–69	12.61 (8.43–17.12)	2.79 (1.26–4.4)	20.69 (10.53–33.33)
70–79	24.66 (14.05–32.14)	6.63 (4.13–12)	30.84 (18.18–45.45)
80 +	36.24 (19.35–50)	17.57 (8.33–34.62)	53.85 (19.05–128.57)
Gender			
Female	5.3 (4.16–5.96)	0.8 (0.46–1.22)	16.06 (8.62–23.53)
Male	5.05 (3.76–6.3)	1.1 (0.84–1.65)	21.21 (15.28–36.17)
Citywide	5.2 (3.95–5.87)	0.98 (0.67–1.53)	18.91 (12.12–28.87)

The rates are estimated for eight age ranges, two genders, and citywide

We also estimate the rates of hospitalization, death, and death in hospital for age ranges and genders. The results can also be found in Table 1. As observed in previous works [17, 24–27, 31–33], the observed rates are larger amongst older than in younger individuals. Moreover, the male population has a higher death rate, although presenting a lower rate of hospitalization than the female population.

When the deaths in hospital reach values above 100% it indicates that the number of daily registered deaths is larger than the number of daily hospitalizations. This may indicate that there are individuals dying before being hospitalized.

During the outbreak of October to December, the hospitalization rate presented lower values than the ones observed during the period 01-Aug-2020 to 05-Oct-2020, whereas the observed death rate remained stable during both periods. Consequently, death rates in hospital increased considerably during the outbreak, which may indicate that only individuals with more severe symptoms are looking for hospital care, decreasing the hospitalization rate.

During the outbreaks of March to May and October to December, the death rate in hospital reached values higher than 40%, which is more than twice the median value obtained during the period 01-Aug-2020 to 05-Oct-2020. This may indicate, as above, that only severely ill people are most likely to search for hospitalization, reducing the observed rate values. The death rates in both outbreaks are considerably different. In the first outbreak the rate reached values higher than 5%, whereas in the second one, it remained around the median value observed during the period 01-Aug-2020 to 05-Oct-2020, i.e., 0.98%. Moreover, the number of individuals tested daily during the first outbreak is much lower than the observed ones during the other two periods.

Thus, based on the insights given by the observed rates of hospitalization and death in Chicago, as well as the number of tests performed, we may infer that, during the outbreak of March to May, the number of COVID-19 infections was considerably underestimated. Notice that we are also assuming that the disease did not change during the period of study, keeping the same rates of severity and mortality.

**Fig. 4:**
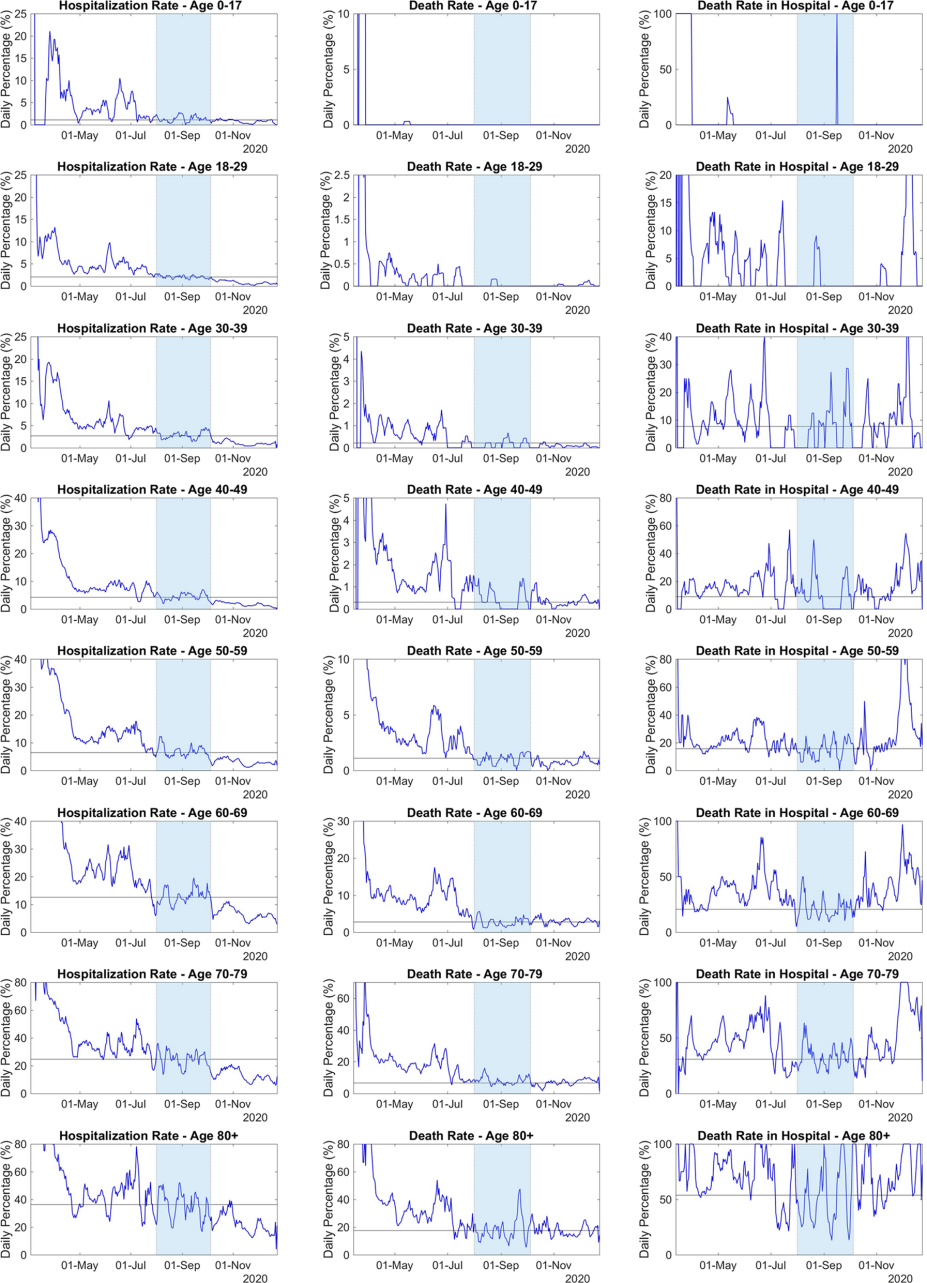
Daily rates of hospitalization (left), death (center) and death amongst hospitalized individuals (right) from 01-Mar-2020 to 23-Dec-2020, in Chicago, for each age range. The dark area shows the period when the rate seems to stabilize, i.e., 01-Aug-2020 to 05-Oct-2020. The horizontal solid lines represent the median values of the rates observed during 01-Aug-2020 to 05-Oct-2020

*Rates by Age Range* In order to analyze the differences between the outbreaks of March to May and October to December in more details, we consider the daily rates of hospitalization, death, and death in hospital by age range. The daily rates can be found in Fig. 4, and the corresponding mean values, as well as 90% CIs are in Table 1.

During the outbreak of October to December, the observed rates of hospitalization, death and death in hospital corresponding to the age range of 0–17 years old remained stable, around the mean values obtained in the period 01-Aug-2020 to 05-Oct-2020. For the other age ranges, the hospitalization rates decreased to values below the observed mean values of the period 01-Aug-2020 to 05-Oct-2020, whereas, the death rates stabilized around the mean, and the death rates in hospital increased considerably above the mean.This is an additional evidence that, during the outbreak, only people with more severe symptoms are looking for hospital care, decreasing the hospitalization rate for all age ranges, but 0–17 years old.

When we look at the rates during outbreak of March to May, for every age range, all the rates of hospitalization and death are considerably higher than the ones observed during period 01-Aug-2020 to 05-Oct-2020 and on the outbreak of October to December. If we assume that the severity and mortality rates of COVID-19 remained constant, we can also assume that from March to May the reports of COVID-19 infections were underestimated. On the other hand, since the death rates during October to December remained around the median values observed on 01-Aug-2020 to 05-Oct-2020, it seems that during this second outbreak underreporting was less likely to happen.

### Underreporting estimation

The aim of the present section is to present the techniques proposed to estimate underreporting by using the observed rates of hospitalization and death. We divide the rates of hospitalization and death by the corresponding values observed during the period when they stabilize around a mean value (period of stability). The results are then multiplied by the reported numbers of infections. More precisely, if $r_H(t)$ and $r_D(t)$ denote, respectively, hospitalization and deaths rates reported on the *t*-th day, using the formulas in Eq. 1, *h* and *d* denote some of the rates of hospitalization and death, respectively, reported in the period of stability, and $\mathcal {I}$ denotes the time series of reported COVID-19 infections, then, the corrected numbers are defined as

2 $$\begin{aligned} \mathcal {I}_H(t-\tau _H)= & {} \mathcal {I}(t-\tau _H)\max \left\{ 1,\dfrac{r_H(t)}{h}\right\} ,~ \text{ and } \nonumber \\ \mathcal {I}_D(t-\tau _D)= & {} \mathcal {I}(t-\tau _D)\max \left\{ 1,\dfrac{r_D(t)}{d}\right\} , \end{aligned}$$

where $\mathcal {I}_H$ and $\mathcal {I}_D$ represent the time series of infections corrected by the hospitalization and death rates, respectively.

In order to avoid indefinite values in the correction procedure, whenever the reported hospitalization or death rate value is zero, we replaced it by the corresponding median value. If the median value is also zero, then, the corresponding formula in Eq. (2) is set to one.

### The epidemiological model

In order to evaluate the impact of underreporting infections on a random mass vaccination strategy, we propose a SEIR-like model [21, 34] to design possible scenarios, considering different situations. The epidemiological model has the following nine compartments: susceptible (S), vaccinated (V), exposed (E), asymptomatic and infective ($I_A$), mildly infective ($I_M$), severely infective or admitted to a hospital ($I_S$), critically infective or admitted to ICU ($I_C$), removed (*R*), and deceased (*D*). We only consider as vaccinated those individuals in the susceptible compartment that receive a vaccine. So, the vaccine efficacy is against infection. If someone already immune or infective receives a vaccine, he or she does not enter to the *V* compartment. The system of ordinary differential equations is the following:

3 $$\begin{aligned}&\dot{{S}} = -{S}(\beta _A {I}_A + \beta _M {I}_M + \beta _S {I}_S + \beta _C {I}_C) - \nu {S} \end{aligned}$$

4 $$\begin{aligned}&\dot{{V}} = \nu {S} \end{aligned}$$

5 $$\begin{aligned}&\dot{{E}} = {S} (\beta _A {I}_A + \beta _M {I}_M + \beta _S {I}_S + \beta _C {I}_C) - \sigma {E} \end{aligned}$$

6 $$\begin{aligned}&\dot{{I}}_A = (1-p)\sigma {E} - \gamma _{R,A} {I}_A \end{aligned}$$

7 $$\begin{aligned}&\dot{{I}}_M = p\sigma {E} - (\gamma _{R,M} + \alpha _S):{I}_M \end{aligned}$$

8 $$\begin{aligned}&\dot{{I}}_S = \alpha _S {I}_M - (\gamma _{R,S} + \alpha _C){I}_S \end{aligned}$$

9 $$\begin{aligned}&\dot{{I}}_C = \alpha _C {I}_S - (\gamma _{R,C} + \delta _D){I}_C \end{aligned}$$

10 $$\begin{aligned}&\dot{{R}} = \gamma _{R,M}{I}_M + \gamma _{R,S}{I}_S + \gamma _{R,C}{I}_C + \gamma _{R,A}{I}_A \end{aligned}$$

11 $$\begin{aligned}&\dot{{D}} = \delta _D {I}_C. \end{aligned}$$

**Fig. 5:**
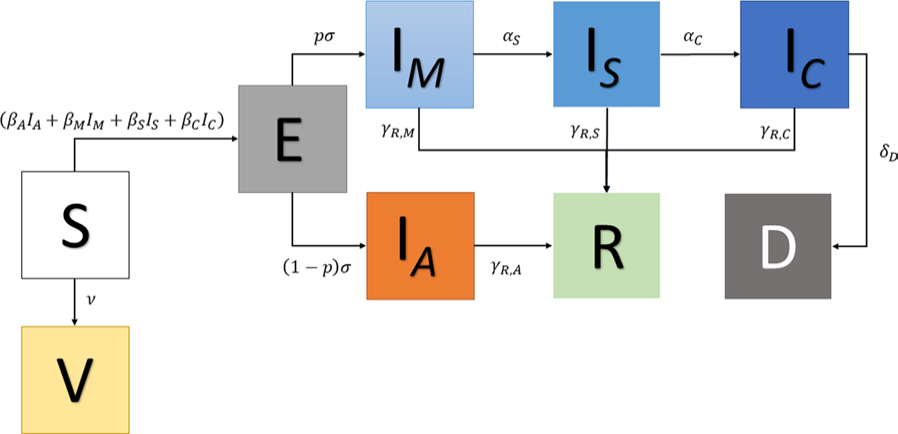
Schematic representation of the SEIR-type model in Eqs. (3,4,5,6,7,8,9,10)–(11)

The schematic representation of the model defined by Eqs. (3,4,5,6,7,8,9,10)–(11) can be found in Fig. 5.

The parameters $\beta _M(t)$, $\beta _S(t)$, and $\beta _A(t)$ are, respectively, the time-dependent transmission rates amongst mildly, severely, critically, and asymptomatic infective individuals. The daily vaccination rate is $\nu$ and the mean time from contagion to becoming infective is $\sigma ^{-1}$. The recovery rate of mildly, severely, critically and asymptomatic infection are denoted, respectively, by $\gamma _{R,M}$, $\gamma _{R,S}$, $\gamma _{R,C}$, and $\gamma _{R,A}$. The parameters $\alpha _S$ and $\alpha _C$ represent the rates of admission to hospital and to an ICU. The death rate of those individuals critically ill is $\delta _D$. Accordingly to [32], only people in critical conditions die by COVID-19, thus, we do not include death rates in the other compartments.

It is worth mentioning that the present model allows the incorporation of virus seasonality since the transmission parameters are time-dependent and adjusted to the daily reported infection.

The parameters $\beta _S$, $\beta _C$ and $\beta _A$ are defined as follows:

$$\begin{aligned} \beta _S = 0.1\beta _M,~\beta _C = 0.01\beta _M,~ \text{ and } \beta _A = 0.58\beta _M. \end{aligned}$$

These definitions mean that, severely, critically, and asymptomatic ill individuals have a reduced capacity of infecting people, due to movement restrictions (in hospital or in an ICU) or by the characteristics of asymptomatic infection [35]. The mean time between infection and onset of symptoms $\sigma ^{-1}$ is set to 5.1, following [28]. The proportion of exposed individuals becoming mildly infective is *p*, which is set to 0.83, following [35]. The recovery rates of mildly, severely, and critically ill individuals are set to one minus the rates of hospitalization, ICU admission and death, respectively. All the asymptomatic individuals will recover in 14 days, thus, $\gamma _{R,A} = 14^{-1}$, which is the average-time until recovery for mildly infective individuals accordingly to [32]. The rates of hospitalization, ICU admission and death are set to the constant values:

$$\begin{aligned} \alpha _S = 0.051,~\alpha _C = 0.39,~\delta _D(t) = \frac{0.186}{0.39}, \end{aligned}$$

where the ICU admission rate was obtained in [36]. The other rates can be found in Table 1. For more details on the numerical implementation of the model and the corresponding parameter estimation technique, we refer to [34].

The model’s solution, simulation, and the estimation procedure were implemented in MATLAB R2019b (The MathWorks, Inc., Natick, USA).

## Results

**Fig. 6:**
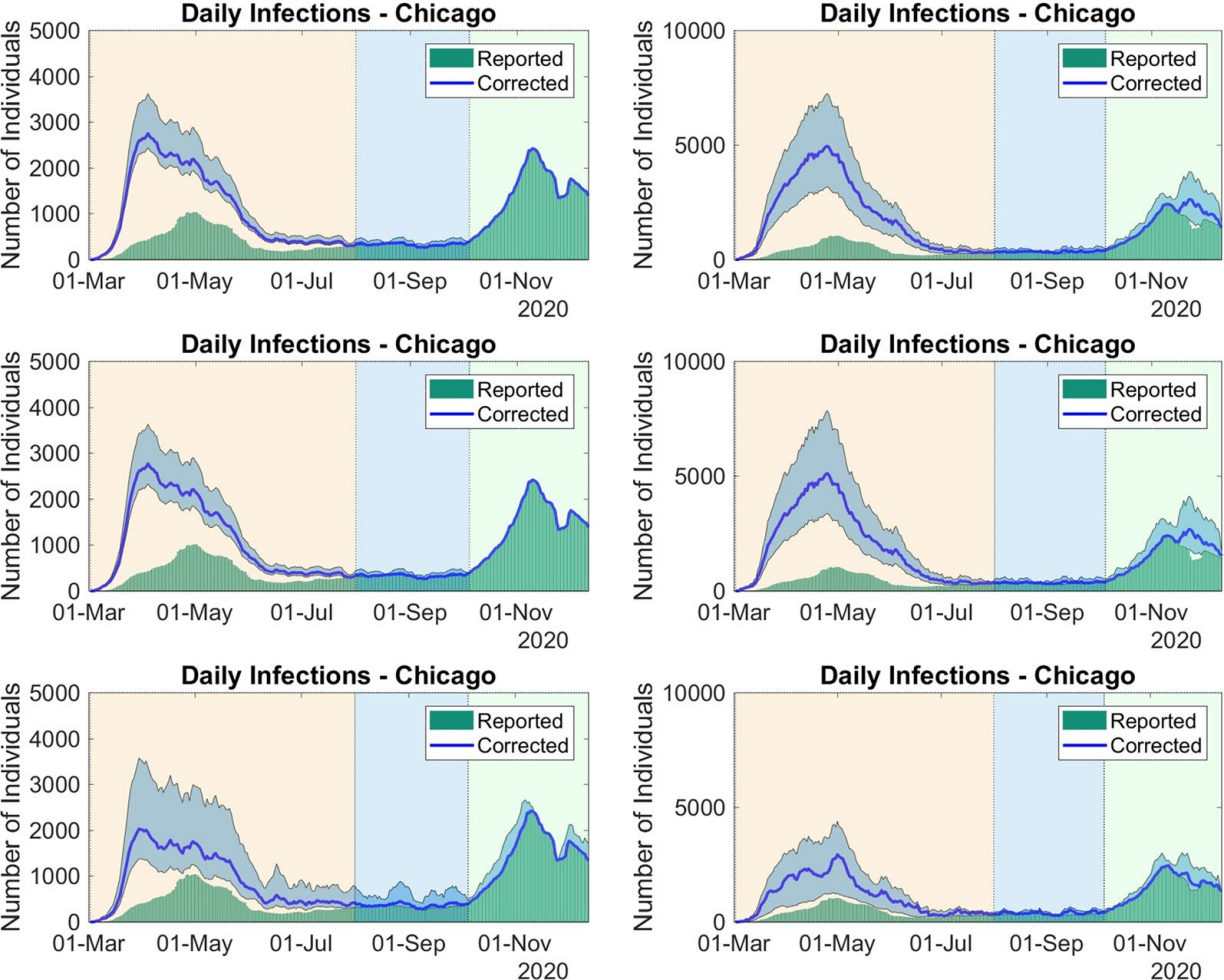
Corrected and reported series of daily infections in Chicago from 01-Mar-2020 to 23-Dec-2020, using the rates of hospitalization (left column) and death (right column) from Table 1. First row uses the daily reports, the second uses daily reports by gender, and the third one uses daily reports by age range. The filled envelopes are 90% confidence intervals (CIs)

*Underreporting Estimation* In order to estimate underreported infections, the formulas in Eq. (2) are used, considering the daily cases of COVID-19. The graphical comparison between the observed and corrected numbers of infections for Chicago can be found in Fig. 6. Table 2 presents the corrected and observed accumulated numbers of COVID-19 infections in Chicago, during the period 01-Mar-2020 to 23-Dec-2020. In order to observe the effect of corrections, we divided the period 01-Mar-2020 to 23-Dec-2020 into three periods, namely, 01-Mar-2020 to 31-July-2020, 01Aug-2020 to 05-Oct-2020, and 06-Oct-2020 to 23-Dec-2020. Additional results considering the data from other places can be found in the Additional file 1.

**Table 2:** Accumulated numbers of corrected and reported infections in Chicago from 01-Mar-2020 to 23-Dec-2020. Corrections use the median values and the 90% CI values from Table 1

Period	By hopitalization rate	By death rate	Observed
Citywide correction			
01-Mar to 31-July	169,126 (149,945–222,446)	290,123 (186,707–423,991)	61,905
01-Aug to 05-Oct	21,772 (21,157–27,321)	22,607 (21,134–30,861)	20,790
06-Oct to 23-Dec	113,992 (113,992–113,994)	127,476 (115,017–167,933)	113,693
01-Mar to 23-Dec	304,890 (285,095–363,762)	440,207 (322,859–622,785)	196,388
Correction by gender			
01-Mar to 31-July	170,244 (143,304–223,130)	297,418 (197,599–453,493)	61,905
01-Aug to 05-Oct	21,994 (21,049–27,231)	23,616 (21,085–33,237)	20,790
06-Oct to 23-Dec	113,561 (113,561–113,712)	129,178 (115,451–178,659)	113,693
01-Mar to 23-Dec	305,798 (277,914–364,073)	450,212 (334,135–665,389)	196,388
Correction by age range			
01-Mar to 31-July	141,280 (97,351–254,365)	168,202 (80,124–264,252)	61,905
01-Aug to 05-Oct	23,998 (21,209–40,851)	25,180 (21,146–31,802)	20,790
06-Oct to 23-Dec	114,188 (113,979–132,578)	118,656 (113,986–146,240)	113,693
01-Mar to 23-Dec	279,466 (232,539–427,795)	312,038 (215,257–442,295)	196,388

Corrections using hospitalization rates present smaller values than the ones obtained with death rates. This can be explained by the considerably larger values of the death rate in hospital observed during the outbreaks of March to May and of October to November. The estimated numbers for 01-Mar-2020 to 31-July-2020 are larger than the ones estimated for other periods, indicating that underreport can be more likely in the beginning of the pandemic. Corrections suggest that, for 01-Mar-2020 to 31-July-2020, the number of infections can be 32–632% larger. For 01-Mar-2020 to 23-Dec-2020, COVID-19 infections can be 10–238% larger. Thus, from 8% to 25% of the population of Chicago could have being infected in the study period, instead of the observed proportion of 7.3%. Such figures are in remarkable agreement with with the seroprevalence study [2], carried out between June and December 2020 in Chicago, which pointed out a seroprevalence of 17.9%.

During larger outbreaks we expect that the stabilization of the daily rates does not occur, as we observed in the case of the daily hospitalization rate during the second wave in Chicago. So, we did not use values from this period in our analysis. On the other hand, the daily death rate remained stable during the second wave in Chicago, suggesting its robustness.

The datasets from NYC do not have daily reports by age range or gender. We considered two different periods to estimate the stable rates of hospitalization and deaths and corrected infections can be found in Additional file 1: Table S.2, representing 7.5–30% of the NYC population, instead of the observed proportion of 4.41%. A seroprevalence study [3] estimated about 1.7 million accumulated infections in NYC by the end of May, which is very similar to our results for the same period, i.e., 1.47 million (1.25 million–2.18 million).

For BA, unfortunately, during the period of study the percentage of positive tests was mostly above 10%, making difficult the empirical analysis. However, we consider the period when the positive rate was below 20%. Additional file 1: Table S.4 presents the estimated rates of hospitalization and death. Death rates for individuals younger than 60 years old are like the corresponding rates observed in Chicago. However, for older individuals in BA, the death rates are considerably larger. Corrections from Additional file 1: Table S.5 suggest infection numbers varying from 3.4% to 303% larger than the notified cases, representing 4.7–18% of the estimated BA population for 2020, instead of the reported 4.53%. Unfortunately, we could not find a seroprevalence study for BA that could be used for comparison.

For MC, we could not identify a period when the rates of death or hospitalization stabilized around mean values. Thus, we used the rates estimated for Chicago to provide corrections. Using the death rates by age-range from Chicago seems to be the more accurate way to estimate underreported cases in other places, since the data from Chicago satisfied the hypotheses made to find stable rates. Corrections are 44–681% larger than the observed cases, representing 5.39–29.1% of the estimated population of MC for 2020. In spite of the issues of the MC data, such estimates are pretty much similar to the seroprevalence of 30.7% (95% CI: 28.3–33.1%) found in the study [5] during December 2020, in the Region Central in Mexico, that includes Mexico City.

In Denmark, from 01-Sep-2020 to 31-Oct-2020, more than 4% of the countrywide population was tested weekly, with positiveness proportions of less than 2%. We used this period to estimate the rates of death and hospitalization in Additional file 1: Table S.7. The corresponding estimations of accumulated cases in 2020 can be found in Additional file 1: Table S.8. Corrections are 23.6–295% larger than the reports, representing 3.35–10.7% of the estimated Danish population in 2020. Such numbers closely agree with the estimated seroprevalence of 4.0% (95% CI: 3.4–4.7%) found by the study [4].

Underreport Impact on Vaccination Scenarios Let us now turn to the impact of underreporting on the capacity of vaccination strategies in reducing hospitalizations and deaths. We consider three different scenarios. The first two consider random-mass vaccination under contained and uncontained spread, whereas in the third an age-range-dependent vaccination is performed under contained spread. The parameters used in these examples are estimated using reports from Chicago and NYC [34].

In all three cases we assume that the proportion of the population in the recovered, exposed or in some infective compartment in the model in Eqs. (3,4,5,6,7,8,9,10)–(11), ranges from 5% to 30%. Moreover, only the amount of 5% is observed in all cases. This means that the probability of vaccinating someone that has already had contact with the virus is proportional to the percentage of the population distributed in the exposed, non-hospitalized and infective, and recovered compartments that were not included in the reports. Thus, in our simulations if 5% of the population was infected, then 100% of the vaccinated individuals were susceptible, whereas, if 30% of the population was infected, then only 73.4% of the vaccinated individuals were susceptible. We also assume that the vaccine is 90% effective, and 0.5% of the population is vaccinated every day, for 150 days. The hospitalization rate also decreased proportionally to the number of underreports.

Under contained spread, the transmission parameter amongst mildly infective individuals is set to $\beta _M = 0.23$. Under uncontained transmission, the parameter $\beta _M$ is set to 0.44. The resulting accumulated numbers during the vaccination strategy, in both situations, can be found in Table 3.

**Table 3:** Accumulated numbers of recovered, vaccinated, hospitalized, and deceased individuals after a random mass vaccination strategy of 150 days, when the proportion of individuals that has already had contact with the virus ranges from 5% to 30% of the population, whereas reports represent only 5%

Contained spread						
Proportion	5%	10%	15 %	20%	25%	30%
Initial recovered	117,704	237,730	357,756	477,782	597,808	717,834
Total recovered	195,683	377,439	542,234	696,487	842,935	983,440
Total vaccinated	1,808,276	1,537,035	1,446,621	1,356,207	1,265,794	1,175,380
Hospitalizations	3192	4847	5993	6625	6892	6893
Deaths	82	120	147	162	167	167
Uncontained spread						
Proportion	5%	10%	15 %	20%	25%	30%
Initial recoverd	117,181	236,675	356,168	475,661	595,154	714,648
Total recovered	428,580	697,122	866,907	1,006,654	1,130,436	1,244,991
Total vaccinated	1,800,249	1,530,212	1,440,200	1,350,187	1,260,175	1,170,162
Hospitalizations	13,107	16,418	17,063	16,554	15,500	14,185
Deaths	200	250	259	251	235	215

The assumed size of this hypothetical population is of 2,693,976 individuals. In Table 3, the numbers in the row *Total Vaccinated* correspond to the vaccinated individuals that were in the susceptible compartment. As the underreported infections increase, the number of effectively vaccinated individuals decreases. The recovered individuals are considered permanently immune. The capacity of vaccination in reducing hospitalizations and deaths is hampered due to underreporting, both under contained and uncontained disease spread. However, if the disease transmission is not under control, then, as underreport increases, the number of hospitalizations and deaths can decrease, indicating the achievement of herd immunity. Therefore, estimating underreporting helps to quantify and explain possible limitations of vaccination strategies.

In the age-range-dependent vaccination case, we use the same vaccination efficacy, and vaccination starts with those aged 80 years or older, then, 10 days after, those individuals aged 70 years or older are included, and so on. Individuals younger than 18 years are not vaccinated. The experiment runs during 150 days, and at each day, 0.5% of the population in each age range included in the strategy for such day is vaccinated. The resulting accumulated numbers can be found in Table 4. The model used to simulate this example is the generalization of the present one as in [23, 34].

**Table 4:** For the (under) reported number of 5%, we present the accumulated numbers of recovered, vaccinated, hospitalized, and deceased individuals after an age-range-dependent vaccination strategy of 150 days, when the proportion of individuals that has already had contact with the virus ranges from 5% to 30% of the population

Proportion	5%	10%	15 %	20%	25%	30%
Initial recovered	123,419	252,838	382,257	511,676	641,095	770,514
Total recovered	367,327	555,216	687,900	801,158	908,024	1,014,017
Total vaccinated	1,808,276	1,537,035	1,446,621	1,356,207	1,265,794	1,175,380
Hospitalizations	6927	8136	7771	6923	5971	5061
Deaths	148	166	152	128	105	84

The accumulated numbers in Table 4 present a similar pattern to those in the previous examples, as expected, illustrating that the underreporting issue can also limit the effect of age-range-dependent vaccination strategies.

## Discussion

This work proposes possible ways to estimate underreported COVID-19 infections, based on daily reported of cases, hospitalizations, and deaths, considering demography. The proposed methodology of correction is then applied to data from Chicago, NYC, BA, MC, and Denmark. Moreover, it estimates the potential impact of underreporting in vaccination strategies by using an SEIR-like model with parameters estimated from real data.

Estimating underreporting in an ongoing epidemic is a hard task, and only a seroprevalence study can address this task appropriately. However, if we can estimate the stable rates of hospitalization and death related to the disease, then we can use reports to estimate the correct number of infections. The major difficulty of this approach is to identify the period when these rates can be observed or approximated. Firstly, we assume that the number of tests performed daily must be sufficiently large, then the number of positive tests must be sufficiently small. Setting up this is subtle, and we must compare the data from different places. For Chicago and NYC, we set that the rate of positive tests must be below 10%, for BA, it was 20%, and for Denmark it was 2%, since we identified, in the corresponding periods, a stabilization of the rates around mean values. For MC, we could not find such period.

For Chicago, NYC, MC, and Denmark during the period of study, corrections suggest that the number of infected individuals could reach 30% of the population of these places, which represents, in some cases, more than six times the reported numbers. These estimated numbers are in remarkable agreement with the estimates from seroprevalence studies carried out in Chicago, NYC, MC, and Denmark during 2020 [2–5]. Moreover, the death rate corresponding to 0.97% estimated in [3] for NYC also agrees with the estimated death rates from Additional file 1: Table S.1, i.e., 1.22% (90% CI: 0.82–1.42%). Such estimates must be considered when evaluating the aftermath of vaccination strategies, since underreporting, as illustrated by numerical examples, can reduce the impact of vaccination in reducing mortality and hospitalization rates. Estimating underreports can be useful, for example, to adjust the daily numbers of given vaccines in order to reach the target of reducing the numbers of infections, hospitalizations, and deaths.

Using age-dependent death rates seems to be a reliable way of estimating underreporting, since such rates can be used even if the age pattern of the infected population changes during the epidemic. Thus, we expect that the more demographic information we incorporate into the death rates, the more reliable are the corrections.

We tested the proposed methodology with data from BA and MC where the positive test proportion was considerably higher than 5% to “stress test” the model, verifying if our premises were still valid when the small positiveness proportion was violated. For MC, we compared our results with the estimates from the seroprevalence study [5] finding again a close agreement between them, in spite of the issues in MC data. This illustrate the possibilities of this approach, since in the developing world seroprevalence studies are generally scarce, and our methodology can shed light on the underreporting issue, providing at least a rough picture of the real number of infections. We believe that our approach represents an accurate alternative to seroprevalence studies that allows anyone who has access to daily reports of infections, deaths and hospitalizations, as well as testing data, to keep track on the underreporting issue. Moreover, for disease surveillance purposes, it can be used as the main underreporting estimation technique or as an independent source of results to validate results from seroprevalence studies.

By considering different vaccination strategies under different disease spread trends, we observe that underreporting can also limit the impact of vaccination in the reduction of hospitalizations and deaths, based on the results obtained with the SEIR-type model in Eqs (3,4,5,6,7,8,9,10)–(11).

## Conclusions

In summary, using the proposed methodology described in Fig. 1 and employing a judiciously chosen data analysis implementation, we estimate COVID-19 underreporting from publicly available data. This leads to a powerful way of quantifying underreporting impact on the efficacy of vaccination strategies. Furthermore, based on the insights given by the observed rates of hospitalization and death in Chicago, as well as the number of tests performed, we may infer that, during the outbreak of March to May 2020, the number of COVID-19 infections was considerably underestimated. Another byproduct of our analysis is that during the outbreak, only people with more severe symptoms were looking for hospital care thus decreasing the hospitalization rate for all age ranges except for the 0–17 years old cohort. Finally, the studies performed for the Chicago case were also conducted for Mexico City, the Province of Buenos Aires, and Denmark resulting in similar conclusions. A natural follow up would be to extend these studies to other metropolitan areas. In the cases of Chicago, NYC, MC, and Denmark, estimated underreported infections closely agreed with seroprevalence studies.

Moreover, by considering vaccination strategies under different disease spread scenarios, using an SEIR-type model, we found that underreporting can also limit the observed reduction in the numbers of deaths and hospitalizations caused by vaccination.

## Supplementary Information

**Additional file 1.** Underreport Estimation and Stable Rates of Hospitalization and Death in Other Locations.

## Data Availability

The data that support the findings of this study are available from the following publicly sources: https://www.data.cityofchicago.org (Chicago), www1.nyc.gov (NYC), https://www.datos.salud.gob.ar (BA), https://www.datos.cdmx.gob.mx (MC), and https://www.covid19.ssi.dk (Denmark). The numerical scripts used to generate corrections and to simulated scenarios can be found in the GitHub repository https://www.github.com/JennySorio/Under_Report.
