# Supplementary material for: COVID-19 underreporting and its impact on vaccination strategies
Source: BMC Infect Dis. 2021 Oct 28;21:1111. doi: 10.1186/s12879-021-06780-7 (PMC8552982; doi:10.1186/s12879-021-06780-7)
Supplement: Supplementary file 1 — Additional file 1. Underreport Estimation and Stable Rates of Hospitalization and Death in Other Locations. [file 12879_2021_6780_MOESM1_ESM.pdf]

Supplentary Materials to: COVID-19 Underreporting and its Impact on Vaccination Strategies

Vinicius Albani,[1*] Jennifer Loria,[2,3] Eduardo Massad,[4,5] Jorge P. Zubelli[6]

[1]Department of Mathematics, Federal University of Santa Catarina,
Florianopolis, Brazil, v.albani@ufsc.br
[2]Instituto de Matemática Pura e Aplicada,
Rio de Janeiro, Brazil, jennyls@impa.br
[3]Universidad de Costa Rica, San José, Costa Rica
[4]School of Medicine, University of São Paulo and LIM01-HCFMUSP,
São Paulo, Brazil
[5]School of Applied Mathematics, Fundação Getúlio Vargas,
Rio de Janeiro, Brazil, eduardo.massad@fgv.br
[6]Mathematics Department, Khalifa University,
Abu Dhabi, UAE, jorge.zubelli@ku.ac.ae

September 16, 2021

1 Under-report Estimation and Stable Rates of Hospitalization and Death in Other Locations

In this section we apply the previous analysis to find stable rates of death and hospitalization using datasets from Chicago to the time series from New York City (NYC), the province of Buenos Aires, and Denmark. All datasets can be accessed on publicly available resources as pointed out below.

New York City In NYC, from 14-May-2020 to 23-Dec-2020, the number of positive tests represents less than 10% of the daily number of tests. In addition, since 14-May-2020, the daily total number of tests is larger than 15,000, i.e., more than 0.18% of the population of NYC estimated for 2020. The reports of daily infections, hospitalizations and deaths from NYC do not include information on gender or age range. Thus, we shall not analyze demographic influence in the rates of hospitalization, death, and death in hospital, which are evaluated using the formulas in Eq. (1). The resulting median values, as well as the corresponding 90% CIs of the rates observed in the time periods 27-Oct-2020 to 10-Dec-2020 and 08-Sept-2020 to 06-Nov-2020 can be found in Table S.1.

Table S.1: Mean values and 90% CIs of the rates of hospitalization and death, as well as the death rate in hospital for the NYC data in two different periods.

Period	Hospitalization Rate	Death Rate	Death Rate in Hospital
27-Oct to 10-Dec	7.13 (5.89–8.49)	1.22 (0.82–1.42)	16.5 (13.1–19.2)
08-Sept to 06-Nov	8.85 (7.34–11.5)	1.22 (0.83–1.91)	14.4 (8.40–21.9)

The hospitalization rates in NYC are higher than those observed in Chicago, but the death rate values are similar. This leads to lower death rates in hospital in NYC than in Chicago. Since the observed death rates in both cities are similar, and since they have a similar demography (censusreporter.org), we have a clear indication of the robustness of the death rates obtained using the proposed methodology.

We corrected the number of infections for NYC using the rates of hospitalization and death from Table S.1 through the formulas in Eq. (2). The results can be found in Table S.2.

Table S.2: Reported and median of corrected accumulated numbers of infections for NYC during the period 29-Feb-2020 to 23-Dec-2020, using the rates from Table S.1. The numbers in the parentheses are 90%CIs. The periods in the table refer to the time span where rates in Table S.1 are observed.

Period	By Hospitalization Rate	By Death Rate
27-Oct to 28-Dec	934,983 (798,548–1,128,399)	1,693,329 (1,465,266–2,492,282)
08-Sept to 06-Nov	771,219 (623,222–909,775)	1,692,389 (1,127,532–2,444,012)
Total number of observed infections for 29-Feb to 23-Dec-2020: 367,930		

The corrected numbers in Table S.2 suggest that a proportion of 7.48% to 29.9% of the population of NYC was infected by COVID-19 during the period 29-Feb-2020 to 23-Dec-2020, instead of the reported percentage of 4.41%. This may significantly affect the vaccination strategy, since, based on such numbers, the chances of vaccinating a susceptible individual can form 100% drop to 73.3%.

Table S.3 shows the accumulated infections during the period 29-Feb to 24-May-2020 and the estimated seroprevalence from [1]. It is worth noticing that the estimated seroprevalence agrees with the present estimated obtained with death rates. It is also worth noticing that, in [1] the estimate infection fatality rate for NYC was of 0.97%, which agrees with the estimates from Table S.1.

Table S.3: Reported, seroprevalence estimates [1], and median of corrected accumulated numbers of infections for NYC during the period 29-Feb-2020 to 24-May-2020, using the rates from Table S.1. The numbers in the parentheses are 90%CIs. The periods in the table refer to the time span where rates in Table S.1 are observed.

Period	By Hospitalization Rate	By Death Rate	Seroprevalence
27-Oct to 28-Dec	723,184 (607,539–876,255)	1,466,678 (1,254,902 2,182,831)	1.7 million
08-Sept to 06-Nov	583,148 (448,116–702,560)	1,465,821 (935,035–2,139,881)	1.7 million
Total number of observed infections for 29-Feb to 24-May-2020: 198,809			

Buenos Aires In Buenos Aires (BA), since 11-Jul-2020, the daily number of tests is larger than 8,000, representing less than 0.05% of the estimated population in 2020, according to www.estadistica.ec.gba.gov.ar. However, since 02-May2020, the proportion of positive tests is always larger than 10%

of the daily total. However, during the period 15-Nov-2020 to 28-Dec-2020, the positive percentage was below 20%, allowing us to choose the period 15-Nov-2020 to 17-Dec-2020 to infer the possible values of the stable rates of hospitalization and death.

Table S.4: Median values and 90% CI (numbers inside the parentheses) of the rates of hospitalization, death and death in hospital observed during the period 15-Nov-2020 to 17-Dec-2020 in BA. The rates are estimated for eight age ranges, two genders, and provincewide.

Age Range	Hospitalization Rate	Death Rate	Death Rate in Hospital
0-17	4.55 (2.88–5.69)	0.13 (0–0.37)	3.03 (0–7.14)
18-29	0.94 (0.55–1.23)	0.08 (0.03–0.15)	10 (3.7–16.67)
30-39	1.56 (1.24–1.97)	0.19 (0.04–0.35)	11.76 (2.13–21.43)
40-49	2.5 (2.1–2.93)	0.55 (0.16–0.69)	22.03 (7.14–26.79)
50-59	4.91 (3.33–6.09)	1.77 (0.82–2.21)	38.39 (16.44–46.03)
60-69	9.93 (6.02–13.12)	7.64 (3.08–9.57)	75.59 (57.66–86.67)
70-79	16.97 (12.82–20.6)	19.01 (7.87–24.3)	110.85 (86.67–137)
80+	24.08 (20–29.86)	32.49 (13.41–42.19)	132.76 (79.78–155.77)
Gender	Hospitalization Rate	Death Rate	Death Rate in Hospital
Female	4.33 (2.95–4.66)	2.88 (1.21–3.44)	67.63 (52.27–75.82)
Male	4.99 (3.23–6.52)	3.94 (1.19–4.46)	73.45 (50.4–81.62)
Provincewide	4.74 (3.04–5.97)	3.39 (1.19–4.01)	71.26 (51.06–79.38)

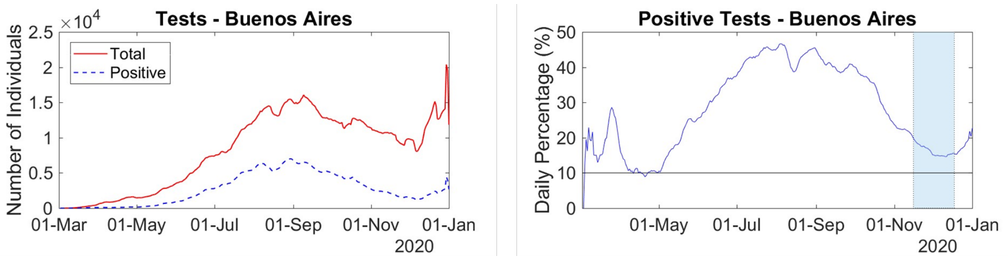

Figure S.1: Left: Daily numbers of tests and positive tests of COVID-19 in BA. Right: The corresponding daily percentage of positive tests. The period is 15Nov-2020 to 17-Dec-2020. The solid horizontal line represents 10%.

Figure S.1 presents the daily totals of performed and positive tests from BA, as well as the daily percentage of positiveness. The median values and 90% CIs of the daily rates of hospitalization and death reported in the period

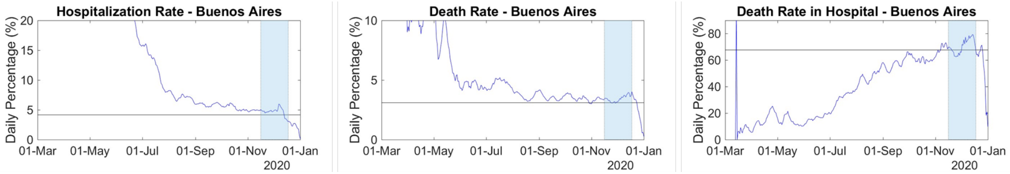

Figure S.2: Daily rates of hospitalization (left), death (center) and death amongst hospitalized individuals (right) from 01-Mar-2020 to 31-Dec-2020, in BA. The dark area shows the period when the rate seems to stabilize, i.e., 15-Nov-2020 to 17-Dec-2020. The horizontal solid lines represent the median of the rates observed during 15-Nov-2020 to 17-Dec-2020.

15-Nov-2020 to 17-Dec-2020 can be found in Figure S.2. Table S.5. presents the median and 90% CIs of the rates reported in 15-Nov-2020 to 17-Dec-2020.

Although the death rates by age range for BA are mostly similar to the ones from Chicago, for individuals older than 60 years old, in BA, the rates are considerably larger, increasing the values of the death rates by gender and for the entire population. In addition, for individuals older than 70 years old, the death rate in hospital attains values larger than 100%, which may indicate death before hospitalization.

Rate	By Hospitalization Rate	By Death Rate	Observed
Provincewide	1,386,759 (1,142,684–2,128,131)	880,739 (820,979–2,383,080)	793,914
Gender	1,400,507 (1214337–2,081,805)	884,337 (824,102–2,362,237)	793,914
Age Range	1,893,104 (1,539,143–2,697,399)	1,057,651 (860,308–3,199,773)	793,914

Table S.5: Reported and corrected accumulated numbers of infections for BA during the period 01-Mar-2020 to 31-Dec-2020, using the median values and 90% CIs (numbers in parentheses) of the rates in Table S.4.

We also estimate the numbers of infections using the rates presented in Table S.4. Corrections suggest that the total number of infections ranges from 3.4% to 303% larger than the reports from 01-Mar-2020 to 31-Dec-2020, representing from 4.7% to 18.2% of the estimated population of Buenos Aires for 2020 (www.estadistica.ec.gba.gov.ar).

Since the death rates for the individuals older than 60 years of age are much larger for BA than those ones for the same age range in Chicago, we used the rates from Chicago to estimate the numbers of infections in BA. In this case we found numbers quite similar to the ones obtained using the original rates from BA.

Mexico City For Mexico City (MC) it was not possible to find a period when the death or hospitalization rates stabilized around a median value. Moreover, the number of tests performed daily was not sufficiently large and, only after 10-Nov-2020, the proportion of positive tests was below 30%. See Figure S.3. The data was downloaded from datos.cdmx.gob.mx and accessed on 07-Jan-2020.

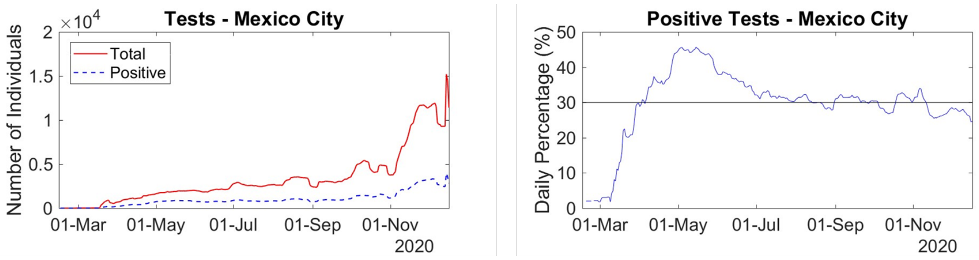

Figure S.3: Left: Daily numbers of tests and positive tests of COVID-19 in BA. Right: The corresponding daily percentage of positive tests. The period is 10Nov-2020 to 23-Dec-2020. The solid horizontal line represents the reference of 30%

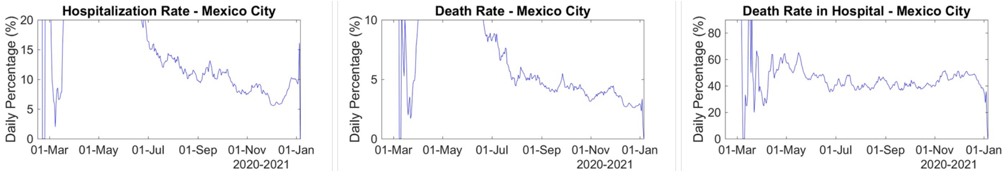

Figure S.4: Daily rates of hospitalization (left), death (center) and death amongst hospitalized individuals (right) from 01-Mar-2020 to 23-Dec-2020, in Mexico City. Apparently, there was no period for the stabilization around the mean or median values for the rates of hospitalization or death.

The graphs of the daily rates of hospitalization and death, as well as the daily rate of death in hospital for the period from 01-Mar-2020 to 23-Dec-2020 can be found in Figure S.4. During this period, the curves of the daily rates of hospitalization and death were decreasing, whereas the daily number of tests was increasing. Only in December the hospitalization rate was increasing. Since July, the death rate in hospital was slightly increasing. In other words, there was no apparent stabilization. Thus, to estimate the number of infections we shall use the rates from Chicago.

Rate	By Hospitalization Rate	By Death Rate
Citywide	782,800 (694,147–1,030,043)	1,793,872 (1,152,847–2,613,067)
Gender	797,762 (670,729–1,042,749)	1,797,170 (1,196,313–2,678,849)
Age Range	673,982 (493,847–1,185,704)	1,576,753 (595,196–2,491,334)
Seroprevalence	2,827,453 (2,606,414–3,048,491)	
Total number of observed infections for 01-Mar to 31-Dec-2020: 342,928		

Table S.6: Reported and corrected accumulated numbers of infections for Mexico City during the period 01-Mar-2020 to 31-Dec-2020, using the median values and 90% CIs (numbers in parentheses) of the rates in Table 1. The bottom row presents the estimation from the seroprevalence study [2].

The estimated numbers of infections, using the rates from Chicago in Table 1, can be found in Table S.6. They suggest that the number of infections varies from 44% to 681% larger than the observed COVID-19 cases. These numbers represent 5.39% to 29.1% of the estimated population of Mexico City for 2020 (cuentame.inegi.org.mx). These results are also in close agreement with the study [2] that founds, in December 2020, a seroprevalence of 30.7% (95%CI: 28.3%–33.1%) in Region Central in Mexico, that includes Mexico City.

Denmark From 01-Sep-2020 to 31-Oct-2020, in Denmark, more than 4% of the countrywide population (worldometers.info) was tested weekly, with positiveness proportions of less than 2% of the sample. For the sake of simplicity, we do not consider gender or age range information in this example. Again, the rates of hospitalization, death, and death in hospital are evaluated using the formulas in Eq. (1), and the resulting median values, as well as their 90% CIs can be found in Table S.7.

Table S.7: Mean values and 90% CIs of the rates of hospitalization and death, as well as the death rate in hospital for Denmark.

Period	Hospitalization Rate	Death Rate	Death Rate in Hospital
01-Sep to 31-Oct	3.78 (2.64–4.82)	0.45 (0.20–0.93)	11.7 (5.26–25.0)

The rates of hospitalization and death in Denmark are smaller than the ones observed in Chicago. Death rates in hospital are also smaller. Such pattern may be linked to a series of factors, such as differences in demography, differences in population behavior, such as larger adherence to disease spread

contention measures, use of masks, keeping social distance, or yet looking for medical assistance in the early stages of the disease.

The corrected numbers of infections for Denmark using the rates of hospitalization and death from Table S.7, using the formulas in Eq. (2), as well as the estimates obtained with the seroprevalence study [3] can be found in Table S.8.

Table S.8: Reported and median of corrected accumulated numbers of infections for Denmark during the period 29-Feb-2020 to 31-Dec-2020, using the rates from Table S.7, and the estimates from the seroprevalence study [3]. The numbers in the parentheses are 90% CIs for the estimates using the rates and 95% CI for the seroprevalence study.

By Hospitalization Rate	By Death Rate	Seroprevalence
216,863 (194,152–292,215)	302,798 (202,836–620,896)	231,688 (196,935–272,233)
Total number of observed infections for 29-Feb to 31-Dec-2020: 166,574		

The numbers from Table S.2 suggest that 3.35% to 10.7% of the population of Denmark was infected by COVID-19 during the period 29-Feb-2020 to 31-Dec-2020, instead of the reported 2.88%, in close agreement with the numbers from study [3], that reported a seroprevalence of 4.0% (95%CI: 3.4%–4.7%).

The data for Denmark was downloaded from covid19.ssi.dk in 25-Aug-2021.

References

[1] Stadlbauer, D., Tan, J., Jiang, K., Hernandez, M.M., Fabre, S., Amanat, F., Teo, C., Arunkumar, G.A., McMahon, M., Capuano, C., Twyman, K., Jhang, J., Nowak, M.D., Simon, V., Sordillo, E.M., van Bakel, H., Krammer, F.: Repeated cross-sectional sero-monitoring of SARS-CoV-2 in New York City. Nature **590**(7844), 146–150 (2021). doi:10.1038/s41586-020-2912-6

[2] Muñoz-Medina, J.E., Grajales-Muñiz, C., Salas-Lais, A.G., Fernandes-Matano, L., López-Macías, C., Monroy-Muñoz, I.E., Santos Coy-Arechavaleta, A., Palomec-Nava, I.D., Duque-Molina, C., Madera-Sandoval, R.L., *et al.*: Sars-cov-2 igg antibodies seroprevalence

and sera neutralizing activity in mexico: A national cross-sectional study during 2020. Microorganisms **9**(4), 850 (2021). doi:10.3390/microorganisms9040850

[3] Espenhain, L., Tribler, S., Sværke Jørgensen, C., Holm Hansen, C., Wolff Sönksen, U., Ethelberg, S.: Prevalence of SARS-CoV-2 antibodies in Denmark 2020: results from nationwide, population-based sero-epidemiological surveys. medRxiv preprint (2021). doi:10.1101/2021.04.07.21254703
